# The radial expansion of the Diego blood group system polymorphisms in Asia: mark of co-migration with the Mongol conquests

**DOI:** 10.1038/s41431-018-0245-9

**Published:** 2018-08-24

**Authors:** Florence Petit, Francesca Minnai, Jacques Chiaroni, Peter A. Underhill, Pascal Bailly, Stéphane Mazières, Caroline Costedoat

**Affiliations:** 1 Aix Marseille Univ, CNRS, EFS, ADES, Marseille, France; 2 University of Sassari, Localita Piandanna, 07100 Sassari, Italy; 3 Etablissement Français du Sang PACA Corse, Marseille, France; 4 Department of Genetics, Stanford University School of Medicine, Stanford, CA USA

**Keywords:** Structural variation, Evolutionary biology

## Abstract

Red cell polymorphisms can provide evidence of human migration and adaptation patterns. In Eurasia, the distribution of Diego blood group system polymorphisms remains unaddressed. To shed light on the dispersal of the Di^a^ antigen, we performed analyses of correlations between the frequencies of DI*01 allele, C2-M217 and C2-M401 Y-chromosome haplotypes ascribed as being of Mongolian-origin and language affiliations, in 75 Eurasian populations including DI*01 frequency data from the HGDP-CEPH panel. We revealed that DI*01 reaches its highest frequency in Mongolia, Turkmenistan and Kyrgyzstan, expanding southward and westward across Asia with Altaic-speaking nomadic carriers of C2-M217, and even more precisely C2-M401, from their homeland presumably in Mongolia, between the third century BCE and the thirteenth century CE. The present study has highlighted the gene-culture co-migration with the demographic movements that occurred during the past two millennia in Central and East Asia. Additionally, this work contributes to a better understanding of the distribution of immunogenic erythrocyte polymorphisms with a view to improve transfusion safety.

## Introduction

**Fig. 1:**
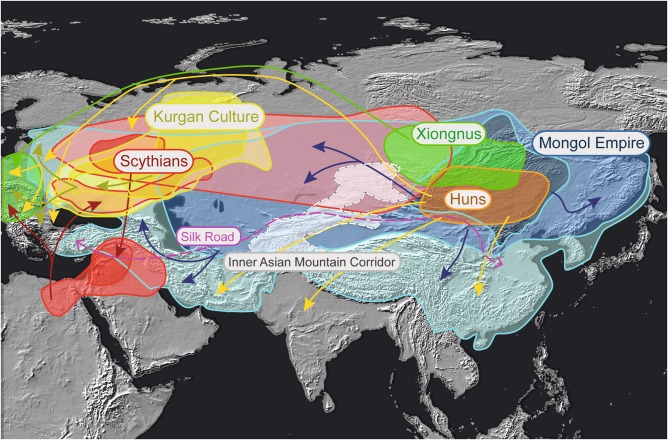
Main migratory and trade movements in Eurasia since the Neolithic. The nomadic mongoloid Xiongnu people established their first empire in the Northern regions of Mongolia between 209 BCE and 93 CE to migrate thereafter to Central Europe, where they mingled with the Franco-Germanic populations. The plotting of arrows indicates general trajectories and do not represent the actual course taken during the movements of populations

Central and East Asia underwent key human expansions since Paleolithic that have contributed to the present-day repartition of many cultural and biological features in Asia and beyond [1–5] (Fig. 1). Notably, archeological and historical records pointed out a reinforcement of population displacements since the Bronze Age (about the second millennium BCE), implying several main Steppe nomadic populations [6]. This period of expansion and exploration is first exemplified by the Indo-European Andronovo culture, which appeared throughout the South Russian steppe and Kazakhstan during the second millennium BCE then diffused eastwards to the Upper Yenisei in the Altai mountains, westwards in the Ural mountains, and southwards until the Amu-Darya basin [7].

The Iron Age Scythians (about 700 to 300 BCE) is another outstanding example of Eurasian expansion. Originating from the Andronovo culture near the Volga river, they occupied the Pontic of the Black Sea (611 BCE), dominated Mesopotamia and Judea, reached Egypt and penetrated several times into Central Europe. When they reigned over the greater part of Central Asia steppes, the Scythians had an important part in the establishment of transcontinental trade, notably the Silk Road [1].

Following the decline of the Scythians, nomadic horsemen peoples flourished in the Altai, near Lake Baikal and in Selenga valley, later migrating to Central Europe where they mingled with the Franco-Germanic populations. Noteworthy were the European Huns led by Attila, who reached Europe at the fourth century CE [8], and the Mongol khans (emperors), who conquered most of Eurasia between the third century BCE and thirteenth century CE. The khans enjoyed strong social prestige, so did their relatives and descendants, resulting in selection pressure by culture for many generations [6].

**Fig. 2:**
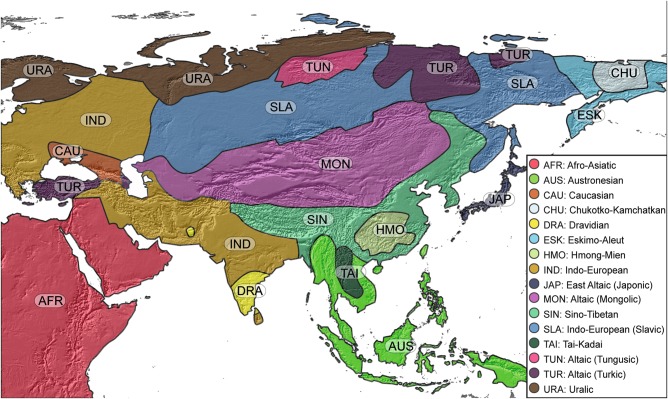
Main pan-Eurasian linguistic families with wide geographical coverage. All isolates and small centers are not represented

The different Steppe nomads were successively Indo-European, Finno-Ugric and Altaic-speakers [6]. Altaic languages include at least Turkic, Mongolic and Tungusic families and are spoken from Turkey and Moldova to Russian Far East [9] (Fig. 2). At genetic level, the Indo-European language has been related to the major Y-chromosome R1-M17 lineage assigned to the centrifugal expansion of the Yamna culture [10]. Altaic-speaking pastoral nomadic populations are mostly carriers of the pan-Eurasian C2-M217 Y-lineage [11, 12]. C2-M217(xM48) patrilineage (embedding C2-M401 and its derivative C2*-ST) is nowadays amplified in Mongols, populations bordering on Mongolia [11, 13–15] and in north Eurasians [5, 16, 17]. Recent refinements of the geographical patterns and age of C2-M217 and its sub-lineages pointed out C2*-ST as part of the founder paternal lineages of all Mongolic-speaking populations, rather than Genghis Khan himself or his relatives [18]. The phylogeographical pattern of the mtDNA diversity in this area is plural and notably witnesses for the same period the admixture of Eastern and Western Asian lineages [19].

Similarly to the uniparental genetic markers, red cell genetic polymorphisms can also trace back past population expansions [20–22] and additionally, be sensitive to natural selection [23, 24]. Notably, the Diego blood group system is a key anthropological marker, since it has evidenced the peopling of the Americas from Asia about 15,000 years ago (reviewed in ref. [20]). Functionally, the Diego blood group system totalizes 22 antigens dispersed at 16 antigenic sites on the erythrocyte membrane glycoprotein band 3. A single substitution (rs2285644, hg19 chr17:g.42328621G>A) defines two alleles, DI*02 and DI*01 responsible for the Di^b^ and Di^a^ antigens, respectively. This transition occurs in the *SLC4A1* gene (17q21.31) which codes for band 3 polypeptide. Noticeably, band 3 is the place of several symptomatic patterns. Some *SLC4A1* substitutions results in preeclampsia, renal tubular acidosis, and Southeast Asian Ovalocytosis (SAO) [25], and as far as the present genetic marker is concerned, the Di^b^ and Di^a^ antigens are highly involved in cases of hemolytic disease of the newborn (HDNB). Hence, such implication suggests a putative contribution of natural selection in the present-day repartition of the Di^a^ antigen amongst populations. But given that its occurrence in Eurasia roughly coincides with the boundaries of the Mongol empires, the actual explanation of the present-day geographical repartition of Di^a^ in Eurasia remains unaddressed.

To tackle this question, we generated original DI*01 data and compared them with previously published data for 75 Eurasian populations screened for the Diego blood group and the Y-chromosome. The effect of the geographical coordinates, linguistics and amount of Y haplogroups and variance on DI*01 distribution received statistical support to discard the role of natural selection and to assume that the Di^a^ antigen has co-migrated with the Mongol expansions.

## Materials and methods

### Allele frequency compilation

Twenty-one Eurasian populations of the HGDP-CEPH panel, representing original data in the present study, were genotyped for rs2285644G>A (Table S1), in addition to four Kyrgyz, four Afghan and eight Pakistani populations previously screened in refs. [15] and [26]. Primers, probes, amplification and capillary conditions were taken from the multiplex SNaPshot array described in ref. [26]. SLC4A1 gene variants have been submitted in public database at https://databases.lovd.nl/shared/transcripts/00019010 (Individual IDs from: 00164740 to 00164758).

We completed with DI*01 allelic frequency data from 38 Eurasian populations from previous studies (Table S1). If only the number of Di^a^ positive phenotypes was mentioned so that it was not possible to distinguish homozygous from heterozygous, the frequency of the DI*01 allele was estimated using Bernstein’s gene-counting method with the formula $p(DI \ast 01) = 1 - \sqrt p [1 - f(Di(a + ))]$, where *f*(*Di*(*a+*)) is the proportion of Di^a^ antigen carriers in the population [27]. By assuming Hardy–Weinberg equilibrium, this method allows calculation of gene frequencies from phenotype frequencies even in the presence of ambiguous cases (e.g., hidden and recessive alleles) but prevents any further selection tests from those frequencies. Data were then plotted onto a single map using the Kriging algorithm of SURFER software version 12.0 (Golden Software, LLC).

For all these same 75 populations, we then merged the DI*01 allele frequency data with Y-chromosome C2-M217 and derivatives frequency data from previous studies (Table S1, ISOGG v. 12.90, March 2017, https://isogg.org/tree/ISOGG_HapgrpC.html).

### Depicting the spread of DI*01 allele across Eurasia

#### Detecting signal of co-migration

To characterize the geographical dispersal of DI*01 across Eurasia, the non-parametric Spearman’s rank correlation coefficient was estimated between DI*01 frequency, latitude, longitude, and frequencies of C2-M217 and C2-M401.

In addition, simple logistic regression models using R software version 3.3.3 (R Foundation for Statistical Computing, Vienna, Austria) and XLSTAT software version 2017.01 were adjusted to explain the presence of DI*01 by geographical coordinates.

#### Analysis of variance influenced by language categories

In addition to allele frequencies, we collected the linguistic affiliation rallied in 7 well-represented categories for accurate statistical analyzes (Table S1) using Ethnologue (https://www.ethnologue.com/), Glottolog v. 3.0 (http://glottolog.org/) and the World Atlas of Language Structures online (http://wals.info/).

One-way ANOVA model was evaluated using the Fisher *F* test to determine whether the amount of information provided by the language factor was significant enough to explain the variance of DI*01 and C2-M217 frequencies.

### Testing the polarity of genetic diversity

With the intention of evidencing a radial expansion from Mongolia to the rest of Asiatic continent, we tested whether the polarity of Y-chromosome genetic diversity agrees with past Mongolian expansions. Indeed, it is accepted that diversity is expected to be the highest at the source population and would decrease as a function of the distance from the source, a.k.a. the founder effect [28–30] or the down-the-line exchange model for archeology records [31]. To this aim, we gathered 234 Y-SNP-STR (Short Tandem Repeats: DYS389I, DYS390, DYS391, DYS392, DYS393, DYS439) haplotypes from 15 Asian populations from Japan to Afghanistan (Table S2).

For each population, we estimated the mean variance encompassed in C2-M217 and C2-M401 individuals, and plotted onto a kriging map. We then estimated the non-parametric Spearman’s rank correlation coefficient between mean C2-M217 and C2-M401 variances with latitude and longitude.

### Testing natural selection

In order to measure the contribution of natural selection in the repartition of the Di^a^ antigen, and thus DI*01, we ran two Hardy–Weinberg (HW) equilibrium tests on rs2285644 genotypes from the 1000 Genome Project database (1KGP) [32] and HGDP-CEPH panel samples including 26 and 31 Eurasian populations respectively, thanks to PLINK version 1.9 [33].

## Results

### Spread directional of DI*01 allele across Eurasia

**Fig. 3:**
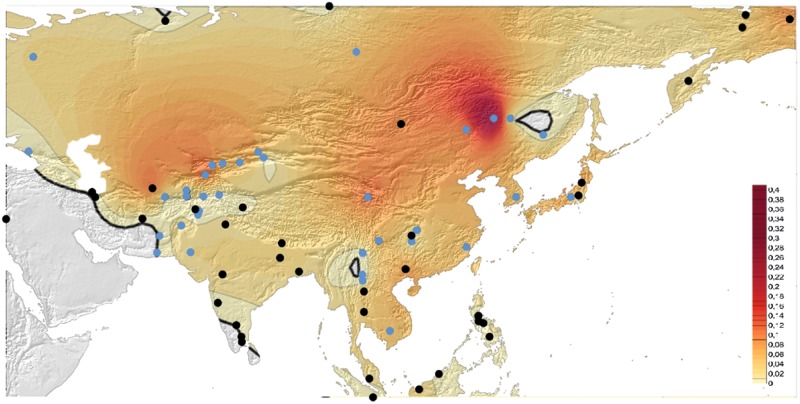
Mapping of DI*01 allele frequency amongst 75 Eurasian populations. Black dots: populations from previous surveys. Blue dots: 21 populations of the HGDP-CEPH panel genotyped in this present study, and in ref. [26] (Table S1). The frequency range 0–0.01 is indicated by marked black lines (in bold for 0, simple for 0.01)

Figure 3 presents the DI*01 allele frequency in Eurasia. Highest frequencies were observed in Mongolia (frequency = 0.098) and Turkmenistan (0.121), followed by Kyrgyzstan (mean frequency = 0.081) and Japan (mean frequency = 0.058). Lower mean frequencies occurred in Cambodia (0.050), China (0.049), North eastern China (Daur and Mongol populations), sporadically in Central (Tu) and western (Uygur) China, and a few pocket areas in South eastern China (Zhuang, Yizu, She and Han), followed by Korea (0.042), Russia and Thailand (0.033). However, the DI*01 allele frequency was null in some populations of southern (Dai, Lahu, Miaozu, Naxi and Tujia) and northern (Hezhen and Oroqen) China. The DI*01 allele was completely absent in Indonesia (Sumatra), Israel (Hebrew), Russia Caucasus (Adygei), and South India (Tamil Nadu, Irula, and Kurumba).

Table 1 presents the correlation test between DI*01 allele frequency, geography, and frequencies of C2-M217 and C2-M401. Results indicated that the amount of DI*01 correlated positively with latitude (*ρ* = 0.291, *p* = 0.011), longitude (*ρ* = 0.297, *p* = 0.010) and amount of Mongolian-origin Y haplogroups (*ρ* = 0.490, *p* = 0.018; *ρ* = 0.428, *p* = 0.042). Similarly, C2-M217 frequency varied as latitude and longitude did (*ρ* *>* 0.520, *p* < 0.011). Alternatively said, frequencies of DI*01 decreased along with amounts of C2-M217 and C2-M401 toward South and West Asia.

**Table 1:** Spearman’s rank correlation *ρ* coefficient between DI*01 allele frequency in 75 Eurasian populations, geography, C2-M217, and C2-M401 haplogroup frequencies

	Latitude (°N)	Longitude (°E)	C2-M217 freq.	C2-M401 freq.
	*ρ* (*p*-value)	*ρ* (*p*-value)	*ρ* (*p*-value)	*ρ* (*p-*value)
DI*01 allele frequency	0.291 (0.011)	0.297 (0.010)	0.490 (0.018)	0.428 (0.042)
C2-M217 frequency	0.520 (0.011)	0.623 (0.002)	—	—

*ρ* estimated value of non-parametric Spearman’s rank correlation coefficient, *p-value* pairwise two-sided *p*-value, *freq.* frequency

In addition, adjusted simple logistic regression model showed that the presence of DI*01 could be explained by longitude (*p* = 0.098), in case significance threshold taken was 0.1 (Table S3).

### Link with cultural linguistic traits

The DI*01 allele distribution portrayed in relation to language showed highest frequencies in Altaic-speakers (mean frequency = 0.071), followed by Eskimo-Aleut, Uralic, Chukchi Kamchatkan and North Caucasian speakers (0.029) from North Eurasia and the Hmong/Tai-Kadai speakers (0.027) from South eastern China. The DI*01 allele is almost complete absent (<0.015) in the Austronesian/Asiatic speakers from Austronesia and the Indo-European family from Middle East and West Asia.

Table 2 presents the effects of language factor in the dispersal of DI*01, C2-M217, and C2-M401. One-way ANOVA shows that the variances of DI*01 allele (Fisher’s *p* = 0.002) and C2-M217 (Fisher’s *p* = 0.001) frequencies were significantly explained by the linguistic background.

**Table 2:** Fisher’s *F* test of the amount of information provided by the language factor in comparison with the mean allele frequency of DI*01, C2-M217, and C2-M401 in 75 Eurasian populations

		DI*01 allele freq.	C2-M217 freq.	C2-M401 freq.
	D° of freedom	*F* (*p*-value)	*F* (*p*-value)	*F* (*p*-value)
Language category	6	3.926 (0.002)	4.155 (0.001)	2.080 (0.070)

*freq.* frequency

### Polarity of genetic diversity with Y-STRs

Table 3 presents the Spearman’s rank correlation results between the mean variance of the Y-STR markers for C2-M217, C2-M401 groups (Table S4) and geographical coordinates. A significant correlation (*p* = 0.010; *p* = 0.042) was obtained only with latitude, so that as variance increased so did latitude. These results were concordant with [12] with significant correlation coefficient with latitude, but our data did not allow to provide the same finding with longitude.

**Table 3:** Spearman’s rank correlation *ρ* coefficient (*p*-value) of the Y-STRs mean variance for the two C2-M217 and C2-M401 population groups

Y-STRs variance	Latitude (°N)	Longitude (°E)
C2-M217 group (15 populations)	0.618 (0.010)	0.104 (0.713)
C2-M401 group (6 populations)	0.829 (0.042)	0.257 (0.623)

**Fig. 4:**
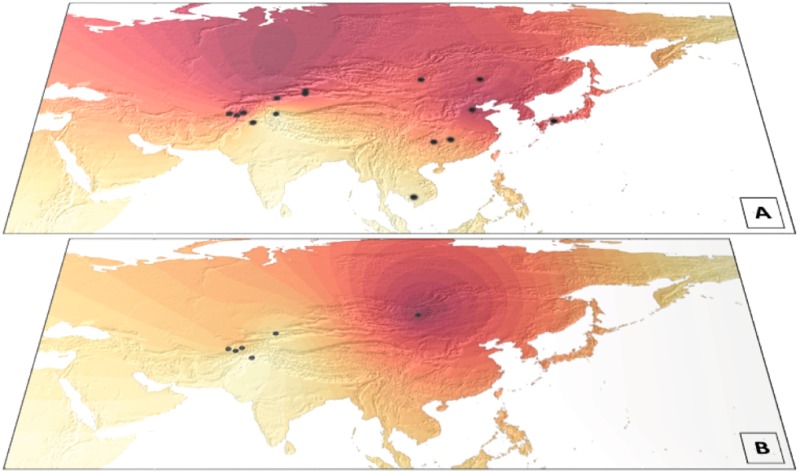
**a**, **b** Mapping of the mean variance of Y-STRs amongst 15 Eurasian populations. **a** Mean variance of C2-M217 carriers from 15 populations (see further details in Table S4). **b** Mean variance of C2-M401 carriers from 6 populations (included in the 15)

Then, we mapped the Y-STRs mean variance Mongolian-origin for C2-M217 and C2-M401 (Fig. 4). Y-STRs variance for C2-M217 was higher in northern than in southern populations, ranging from 0.431 (Northern Hans in China), to less than 0.100 in the Hazaras from Pakistan (0.096) and even 0.000 in Cambodia and for the Burusho population in Pakistan (Fig. 4a). Figure 4b shows a hotspot of mean variance of C2-M401 in Mongolia (0.105), decreasing towards Pakistan.

### Testing natural selection

Runs of HW equilibrium test allowed to discard natural selection at the rs2285644 locus in all populations from the 1KG project and HGDP-CEPH panel (*p*_*χ*2_ = 1) but 2: Han (*p*_*χ*2_ = 0.002) and Tu (*p*_*χ*2_ = 0.027) from China (Table S5).

## Discussion

### Historical marks of co-migration from Mongolia and the Diego-Y-chromosome duet in Central Asia

The C2-M217xM48 male-lineage, which amplified in Altaic-speaking pastoral nomadic groups [12, 18] signposts significantly the East-to-West Mongolian expansions which may have also towed the Diego blood group polymorphism across Eurasia. Additional support of our assumption is given by the contrasting pattern observed in India, where DI*01 has been only detected in the northern populations (Bhil Madhya, Rajbanshi Bengali, Bihar, Oraon, and Punjab) and further North of India (Nosherpa, Nepal, Sindhi, Balochi and Hazara, Pakistan) populations, with null DI*01 frequencies in the South (Tamil Nadu, Irula, and Kurumba populations) and South-East. This pattern could be potentially explained by the conquests in India following the Mongolian expansion. The Mughal—or Mogul—Empire (1526–1857) was founded in northern India in 1526 by Babur, descendant of Tamerlane (1336–1405), first ruler in the Timurid dynasty, commonly considered nomadic Turco-Mongol conqueror of Eurasian Steppe [34] and of Genghis Khan. At the end of Akbar’s reign in 1605, the Empire stretched from Punjab, Bengal to Gujarat. Some territories were notwithstanding conquered in a southerly direction in the mid-seventeenth century [35] leaving no visible genetic traces using our data.

Several independent genetic features, such as the *EDAR* and ADH1B*47His alleles whose fixation would have started through positive selection at the earliest stages of Neolithic, also showed a clear geographical cline amongst East Asians due to human expansion [36, 37], but preceding the Mongols.

Worldwide, very few non-Mongol-origin individuals have been identified as carrying Di^a^ antigen [38–40]. Sporadic occurrence of DI*01 in population bordering on the Mongolian expansion, such as in Poland that has been invaded by the Mongolian Tartars during the thirteenth century and between the fifteenth and seventeeth centuries [41] and in Afghanistan and Pakistan populations close to the Hazara [26], might originate from admixture.

Thus, we relied on the sharp cut signal of the Y-chromosome to point out the polarity of the Di^a^ expansion. The pattern is consistent with an East Asian source that have expanded through the several Mongol expansions, strengthened by linguistic background and also in concordance with the diffusion of a package of East Asian mtDNAs as presented in ref. [19].

### Could the presence of Di^a^ in continental Asia populations also be attributable to natural selection?

Given the role of band 3 onto which the Diego antigens are erected, natural selection is expected to have acted at some point. Noteworthy is the association of the Di^a^ antigen with severe or even fatal HDNB, that would have strongly disrupted the penetration of the DI*01 allele in a Di^b^ world. As previously described in Native Amerindians [20], the role of natural selection at the rs2285644 locus in continental Eurasian populations do not receive support from the present data.

### In-depth examination of the DI*01 allele

To attempt to spot, within the *SLC4A1* gene, alleles usually associated with the DI*01 allele, we have estimated by using PLINK version 1.9, the allele frequencies of SNPs belonging to this gene and available in the 1KGP. Positive and significant Spearman’s rank correlation coefficients were found between the distribution of rs2285644 (derived nucleotide: A) and that of SNP mutations previously known to be responsible for preeclampsia, renal tubular acidosis, and mostly SAO [25]; the two latter being usually associated [42]. To go further, among the 12 mutations known to be responsible for the SAO [43], we have identified 4 SNPs: rs5036 (hg19 chr17:g.42338945T>C, ancestral nucleotide: C, responsible for band 3-Memphis non-synonymous polymorphism [44]), rs16940582 (hg19 chr17:g.42339745C>T, derived nucleotide: T), rs16940585 (hg19 chr17:g.42339762G>A, derived nucleotide: A) and rs2521602 (hg19 chr17:g.42336424G>A, derived nucleotide: A), whose distribution was always, worldly and only at Asiatic scale too, correlated with rs2285644 (A). These different results could be considered as a track of association between Diego polymorphism and SAO. Taking into account the sparse geographical coverage of the sample used (10 Asian among 26 populations), the results should be confirmed by additional studies.

To conclude with, the aim of this study was to disentangle the geographical distribution of the DI*01 allele of the Diego blood group system in Eurasia. Our data demonstrated large variations in frequency ranges with a hotspot in Mongolia, and a significant positive correlation with geographical coordinates. Our findings suggested that DI*01 carriers could have crossed into West Asia from Mongolia as indicative of a striking historical and linguistic event: the expansions of Altaic-speaking pastoral nomads from Mongolia with high reproductive success. In a broader context, the description and understanding of the present-day geographical distribution of red cell phenotypes come under a multidisciplinary approach as herein developed. Beyond the anthropological interest, the elucidation of well-circumscribed areas of immunologic polymorphisms is a crucial step to ensure in blood transfusion safety, and this is, in our opinion, what the present study contributes to.

## Electronic supplementary material

Table S1

Table S2

Table S3

Table S4
